# A double layer FSS filter for sub-THz applications

**DOI:** 10.1038/s41598-021-99256-2

**Published:** 2021-10-05

**Authors:** A. Ghavidel, M. Kokkonen, S. Myllymäki

**Affiliations:** Microelectronics Research Unit, University of Oulu, 90014 Oulu, Finland

**Keywords:** Engineering, Electrical and electronic engineering

## Abstract

This work presents the simulated and measured performance of single- and double-layer frequency selective surface filters for operation at sub-THz frequencies (250 GHz center frequency). They were composed of concentric square loops with a split as a unit cell resonator on top of a low dielectric permittivity, low thickness material (RT5880). Both a single layer filter and a cascaded two layer filter with varied distances were investigated. The simulated bandwidth for the cascaded filter was 27 GHz and 16 GHz and 9 GHz bandwidth measured with a THz-TDS and microwave system.

## Introduction

The terahertz band (0.1–10 THz) and especially the sub-THz (0.1–0.3 THz) band are new unallocated radio bands offering new possibilities for future sixth generation (6G) communications, sensing, imaging and joint communication-sensing^1–5^. Applications in telecommunication, imaging and spectroscopy benefit from the increased bandwidth, small wavelength and high penetrability of non-ionizing radiation^3,6–9^. In localization, sensing and imaging applications, the millimeter (mmWave) technologies offer greater accuracy, which promotes the emergence of new applications^5,10–14^.

For many transceivers it is critical to have good signal-to-noise (SNR) and signal-to-interference-plus-noise ratios (SINR) which can be improved in the THz band with a Frequency Selective Surface (FSS) instead of using a common circuit filter. A FSS filter consists of periodically arranged conductive surfaces having a variety of specific geometries on top of the dielectric surface^15^. Compared to a common circuit type filter, a FSS filter benefits from lower costs, lower electrical losses and it is more compact in size which allows cheaper and easier prototyping^16,17^.

FSS technology has been exploited for different applications, such as controllable “smart” surfaces, miniaturized cavity resonators, wave-guide structures, angular-independent surfaces, absorbers, biomedical devices, terahertz switches, fluid-tunable frequency-agile materials, phase modulation, the design of antenna radomes, dichroic surfaces for reflectors and sub-reflectors of large aperture antennas, diplexers, beam splitters and many other applications^18–28^.

For many applications, the bandwidth of the filter is important and, in the FSS, bandwidth depends on the unit cell and substrate material^29^. The cascade layers of the FSS have been proposed to be used to increase the operation bandwidth because they lead to a flatter, wider and more stable insertion loss^30,31^. Misalignment of the cascaded layers has been investigated and found to lead to decreased performance (transmittance and bandwidth)^31^. The overall frequency response of the FSS layer filter, such as its bandwidth, transfer function and its dependence on the incidence wave angle and polarization, are determined by the grating (inter-element) spacing besides the unit cell parameters^30^.

The FSS layer typically has grating lobes which are the result of electromagnetic diffraction and spacing between unit cells^18^. Having a suitable spacing between the unit cells can increase the bandwidth and provide a stable resonance frequency for the filter with respect to the incident angle variations^18^.

Telecommunication needs Split Ring Resonator (SRR) filters with very wide bandwidths, which cannot easily be met at present. This raises the need to investigate one and two FSS layer bandwidth capability and insertion loss parameters at a particular Sub-THz frequency band (220 GHz to 330 GHz) for the upcoming 6G telecommunication systems.

Previously, a double metamaterial layer resonator with semiconductor (GaAs) material has been studied by tuning the distances of the layers, for THz-TDS with an operation frequency of 0.35 THz^32^. Also, a similar study has been conducted on a double layer cross frequency selective surface (FSS) unit cell using only metal material (aluminum), fabricated by femtosecond laser machining, for an operating frequency of 0.55 THz^33^.

In this work, we present a cascaded FSS bandpass filter using low permittivity thin Printed Circuit Board (PCB) operating at 250 GHz. Simulations and design for a single and a double layer FSS using square loop shaped Split Ring Resonators (SRR) are described and analyzed in “Design and simulation of sub-THz bandpass filter” section. In “Experimental results” section, experimental achievements using two different free space measurement methods are presented and discussed. Comparison of the simulations and measurement results are discussed in “Comparison between the simulation and experiments” section. In “Conclusion” section conclusion to this study is offered.

## Design and simulation of sub-THz bandpass filter

The firsts step to design the FSS filter was to choose the geometry of the unit cell and the substrate material. Also, an equivalent circuit model of the FSS using lumped elements (L, C, and R) was designed to analyze the frequency resonance of the filter^34^. Finally, the unit cell matrix (or array) was designed as an FSS layer. A cascaded FSS filter was then formed by placing a second FSS filter on top of the first one with a spacing of 1.5λ.

### Circuit model of single unit cell

For this study, a square loop shape SRR was considered with regard to its bandwidth potential and the fact that it has previously been investigated using both analytical expression and a circuit model^35,36^.

**Figure 1:**
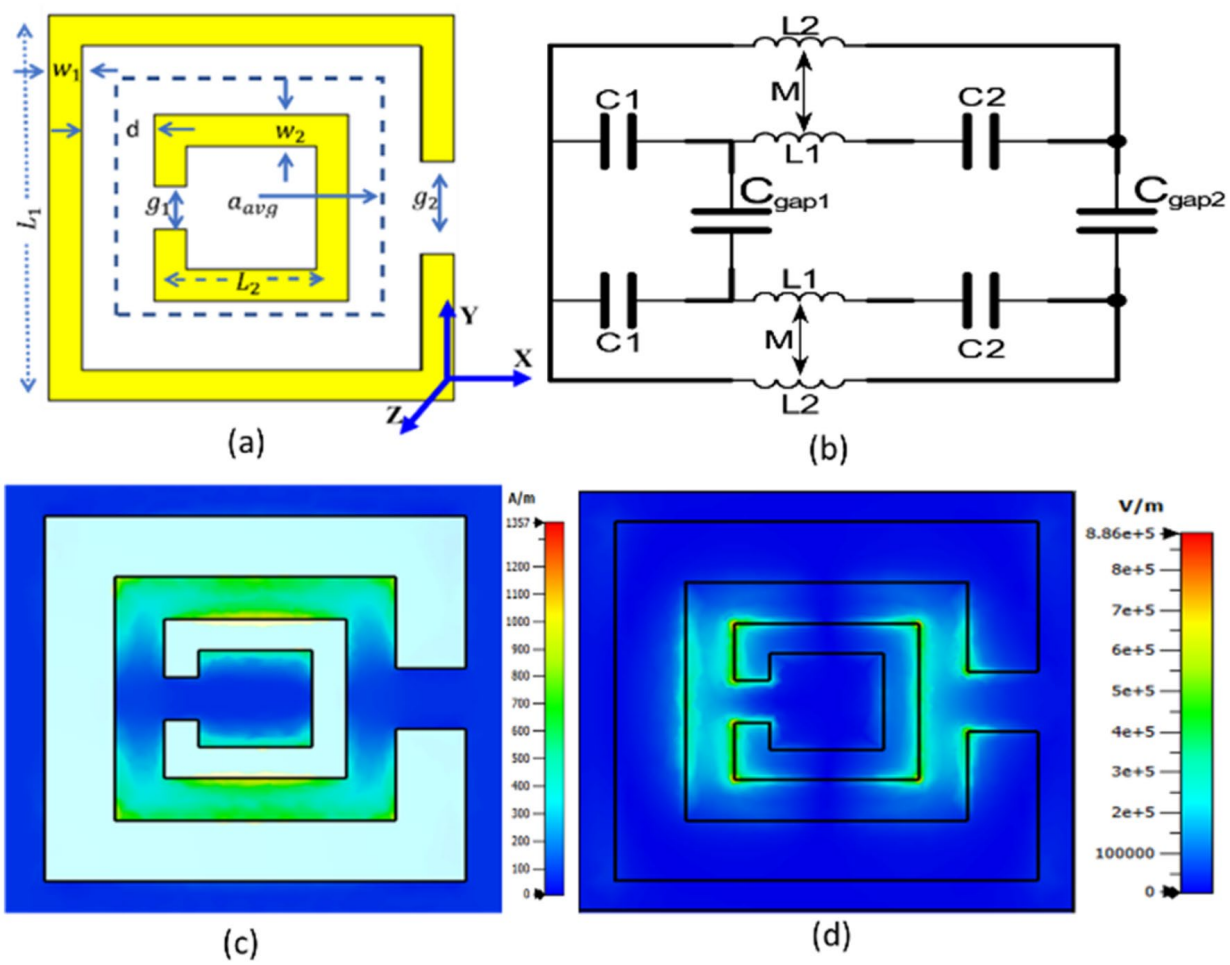
(**a**) Schematic of the single SRR (values are presented in the Table 1), (**b**) equivalent circuit model of the SRR for TEM mode, (**c**) Simulated magnetic field was coupled in the y-direction and (**d**) Simulated electric field was coupled in the x-direction.

The SRR with an opposing split in the loop was designed for a 250 GHz center frequency on a PCB (RT5880, ε_r_ = 2.2)^37^ using a full wave simulator (CST)^38^. The schematic of the SRR is shown in Fig. 1a and parameters for the unit cell are listed in Table 1. An equivalent circuit model of the SRR for Transverse Electro Magnetic waves (TEM) mode with respective symbols for capacitance C, inductance L and gap in the ring C_gap_ where index 1 represents the outer ring and index 2 the inner ring is shown in Fig. 1b. Between the rings there is mutual magnetic coupling M which happens due the metal also acts as a coil and its magnetic field couples between the coils. Simulated magnetic and electric fields are in Fig. 1c,d. The H-field was coupled in the y-direction and E-field in the x-direction when the plane wave passed through the SRR in the z-direction.

**Table 1:** Unit cell geometry dimension for 250 GHz.

Parameters	w_1_	w_2_	g_1_	g_2_	L_1_	L_2_	t
Dimension (µm)	100	50	70	100	600	260	50

### Modeling of two cascaded unit cells

**Figure 2:**
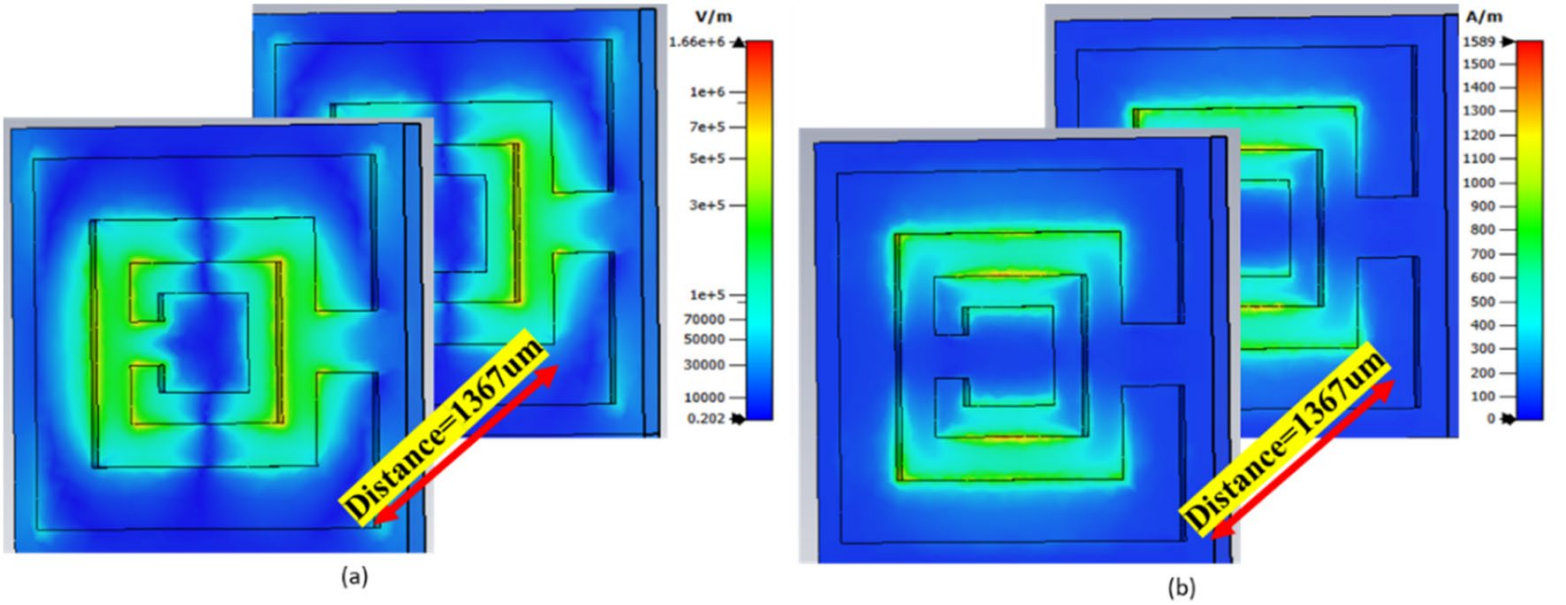
Simulated (**a**) Electric field and (**b**) Magnetic field. Fields’ direction and the coupling are similar to the single unit layer, the difference to the single unit was the amplitude of the fields.

Two-unit cells were placed at a distance of 900 µm from each other and their simulated electric and magnetic field amplitudes are presented in Fig. 2. A plane wave traveled through the layers as in the single layer case and E- and H-fields are presented in Fig. 2a,b. Having a cascaded structure did not alter the fields’ coupling directions, it only changed the field strengths; E-field (from 8.8 × 10^5^ to 1.6 × 10^6^ V/m) and H-field (1357 to1589 A/m).

**Figure 3:**
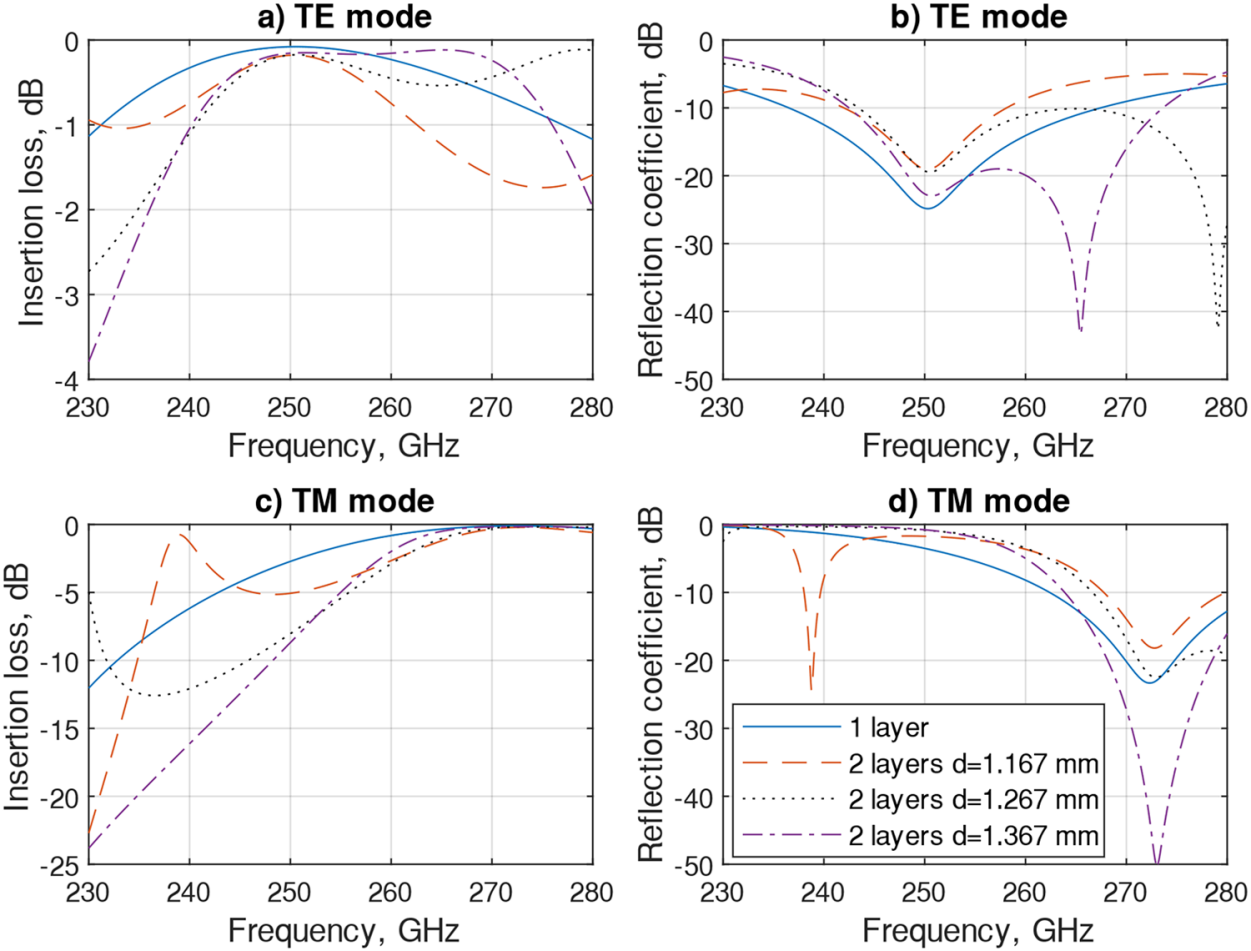
Simulated unit cell S-parameters: for TE polarization, (**a**) insertion losses and (**b**) reflection coefficient and for TM polarization, (**c**) insertion losses and (**d**) reflection coefficient. Filter was designed for 250 GHz TE polarization.

**Figure 4:**
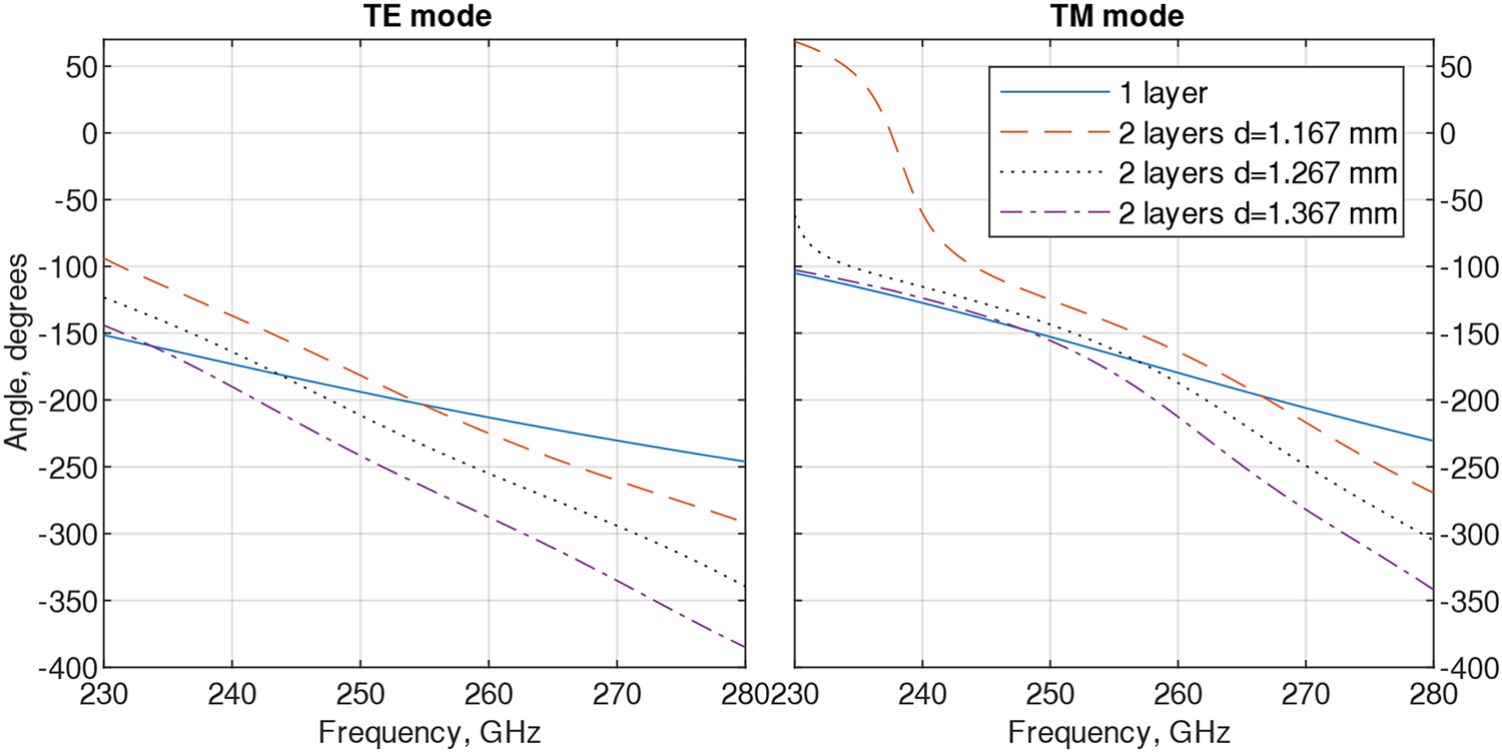
Simulated phase for both TE and TM polarizations.

Simulated S-parameters, insertion loss (S21) and reflection coefficient (S21) for Transverse Electric wave (TE) and Transverse Magnetic wave (TM) modes for the broad band 230–280 GHz for single and two-layer with three spacings were investigated and results are shown in Fig. 3. For TE mode, a single unit cell performed with a lower than − 10 dB reflection coefficient (S11) from 237 to 265 GHz and with insertion losses (S21) approximately 0.1 dB. The two-layer FSS, showed a − 10 dB reflection coefficient with insertion losses around 0.12 dB at 242 GHz to 272 GHz. There was a second resonance presented with 1.267 mm and 1.367 mm distances at frequencies 265 GHz and 278 GHz, respectively. Cascading the unit cells mutual coupling impacted the S11 scattering parameter. On increasing the spacing between layers, the bandwidth of the SRR filter decreased, and vice versa. As seen in Fig. 3b, the S11 with a spacing of 1.267 mm showed more mutual coupling than at 1.367 mm spacing and the bandwidth was wider. For TM mode, the frequency has been shifted to 272 GHz showing around − 20 dB reflection losses at all but the 1.367-layer distance, S11 was − 50 dB and insertions losses for all cases were near 0 dB. Furthermore, the impact of the second layer on the simulated phase is seen in Fig. 4 and it shows some phase deviation between one and two-layer SRR filters at both modes.

Parameters regarding the FSS bandwidth, S parameters, reflection coefficient, insertion loss, phase and distance for the TE mode are shown in Table 2. Changing the spacing between two layers can impact operation parameters including bandwidth (BW) and insertion loss and phase difference. For example, by increasing the spacing the bandwidth became wider, 27 GHz, but insertion loss was increased as well. On the other hand, reducing spacing (1267 mm, optimized structure), the bandwidth decreased to 18 GHz, but insertion loss reduced. By decreasing the spacing between the layers even further, the BW decreased to 15 GHz with no visible changes in the insertion loss.

**Table 2:** Simulated unit cell performance parameters for TE mode.

BW (GHz)	S11 (dB)	S21 (dB)	Phase at 250 GHz (degrees)	spacing between the layers (mm)
10	− 25	0.1	− 194	–
27	− 23	0.2	− 242	1367
40	− 20	0.25	− 221	1267
/	− 19	0.25	− 182	1167

### Full array modeling analysis

**Figure 5:**
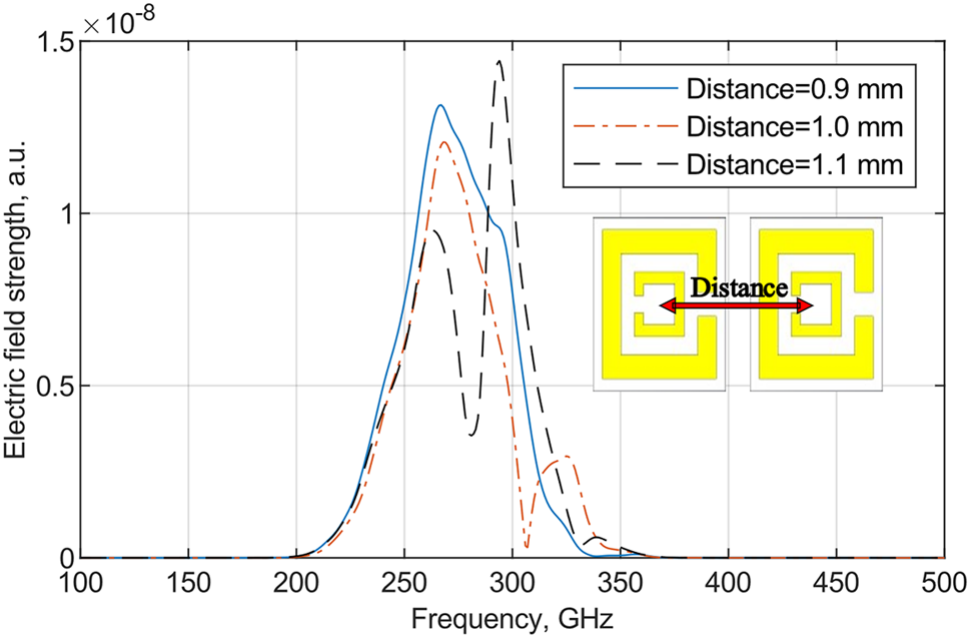
Simulated electrical field strength at front of the FSS illuminated by TE mode planewave from the rear side, with three different interelement spacings. Change in inter element spacings leads to a change in the bandwidth.

The full array was modelled with 32 × 30 elements on a planar lattice with an inter element distance of λ/2 and a TE mode planewave illuminating the filter from the rear side. An electric probe was placed in front of the FSS to measure the electric field strength after it had passed through the FSS. The periodic lattice caused phenomena e.g., surface wave, surface impedance and mutual impedance, besides the resonant phenomena. Interelement spacing could control unwanted grating lobes, which had the potential to degrade the performance. Inter element distances were simulated for three distances and results are shown in Fig. 5. Decreasing the distance, the bandwidth was decreased, and vice versa.

## Experimental results

In this section, the FSS filter was fabricated, and its electrical performance was measured using two different methods, THz time-domain spectroscopy (THz-TDS) and microwave systems. THz-TDS was used to measure transmittance and time delay and the microwave system was used to measure the S-parameters.

### Fabrication of FSS filter layer

**Figure 6:**
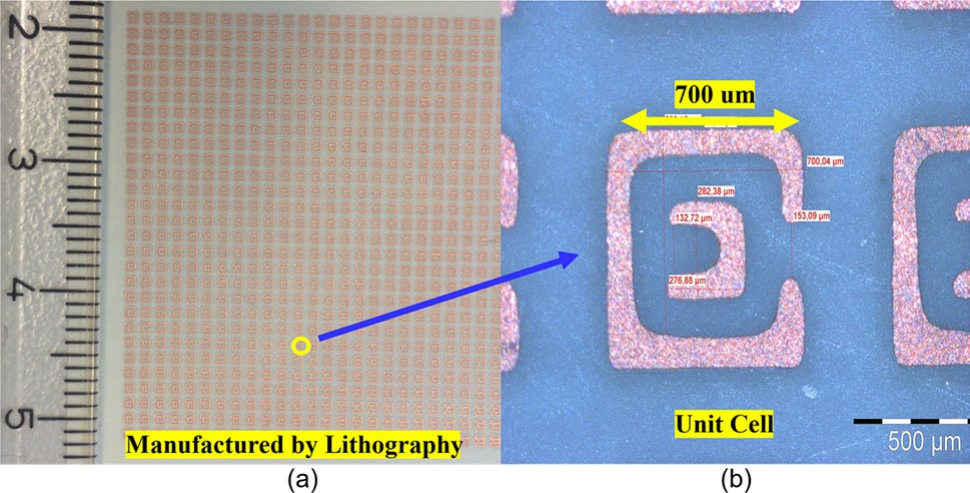
Picture of fabricated FSS filter having 32 × 30 units on area ~ 3 × 3 cm^2^, fabricated by lithography and zoomed image to the single unit cell which shows some erosion from lithography.

The FSS filter was fabricated using low permittivity material (RT5880, ε = 2.2) and a photolithography method and its picture is shown in Fig. 6. The fabricated FSS layer was composed of 960-unit cells, Fig. 6, (a) left side and the picture of a square loop SRR, right side Fig. 6b. The close up image shows some additional erosion which came from the lithography method, as expected.

### Measurement using THz time-domain spectroscopy

**Figure 7:**
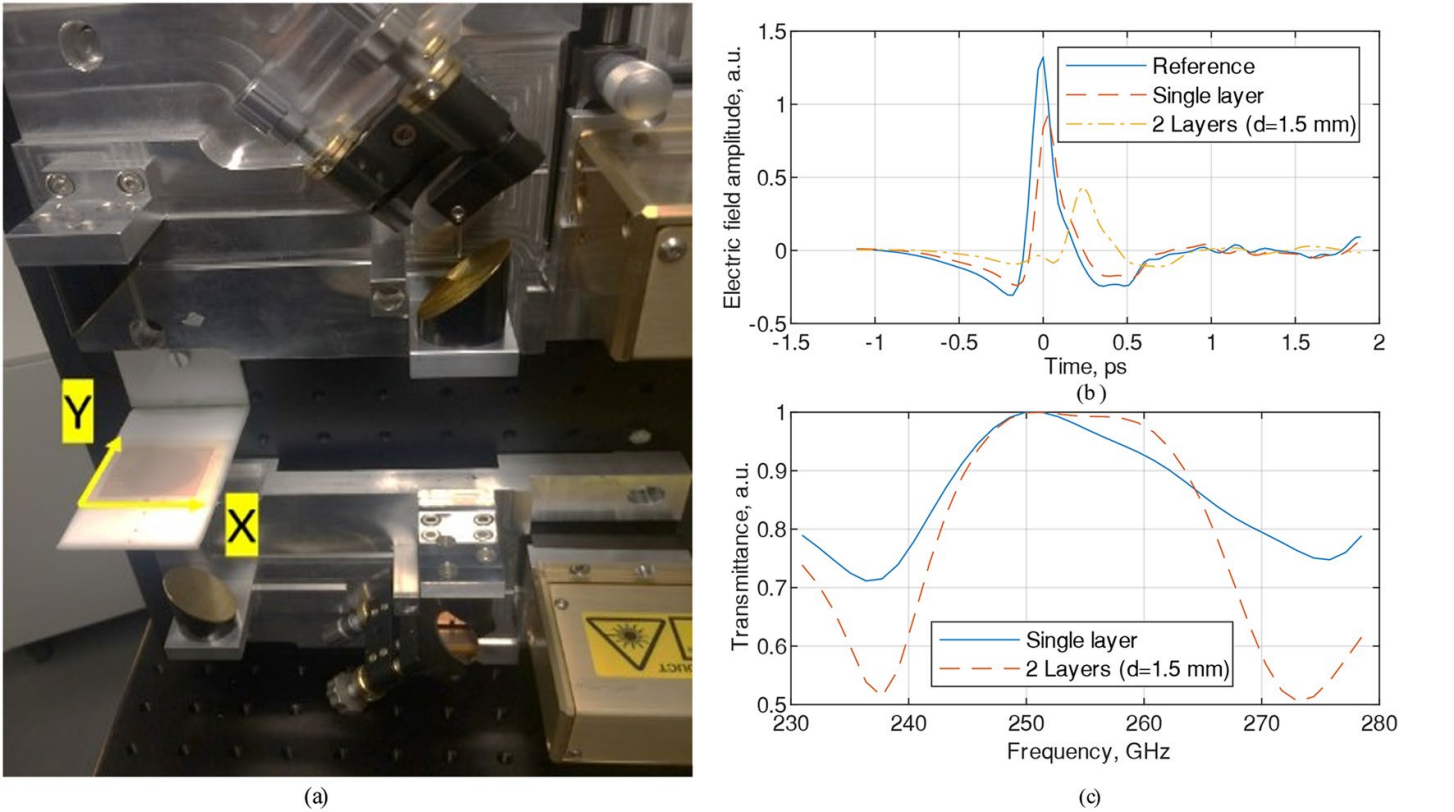
(**a**) TeraPulse 4000 Spectroscopy system for measuring FSS filter, measured and normalized (**b**) measured pulse response of electric field amplitude where the refence signal was measured without the FSS layer and (**c**) measured signal transmittance of single- and double-layer unit cell.

A TeraPulse system (TeraPulse 4000, Teraview UK)^39^, Fig. 7a, was used to measure the FSS filter’s impact on time-delay and transmittance and the results are shown in Fig. 7b,c. The single layer FSS filter was placed between two mirrors and the thickness of the FSS created a time-delay (around 0.1 picosecond) and reduced the amplitude to 0.9 compared to the reference signal amplitude (12.5). With a second layer, the time-delay increased to 0.5 ps and reduced the transmittance amplitude to 0.43. It was observed that each layer reduced the transmitted wave amplitude by around 30%. The bandwidth was measured to be ~ 12 GHz with one layer and ~ 16 GHz with two layers at 95% transmittance level.

### Measurement set up using microwave system

**Figure 8:**
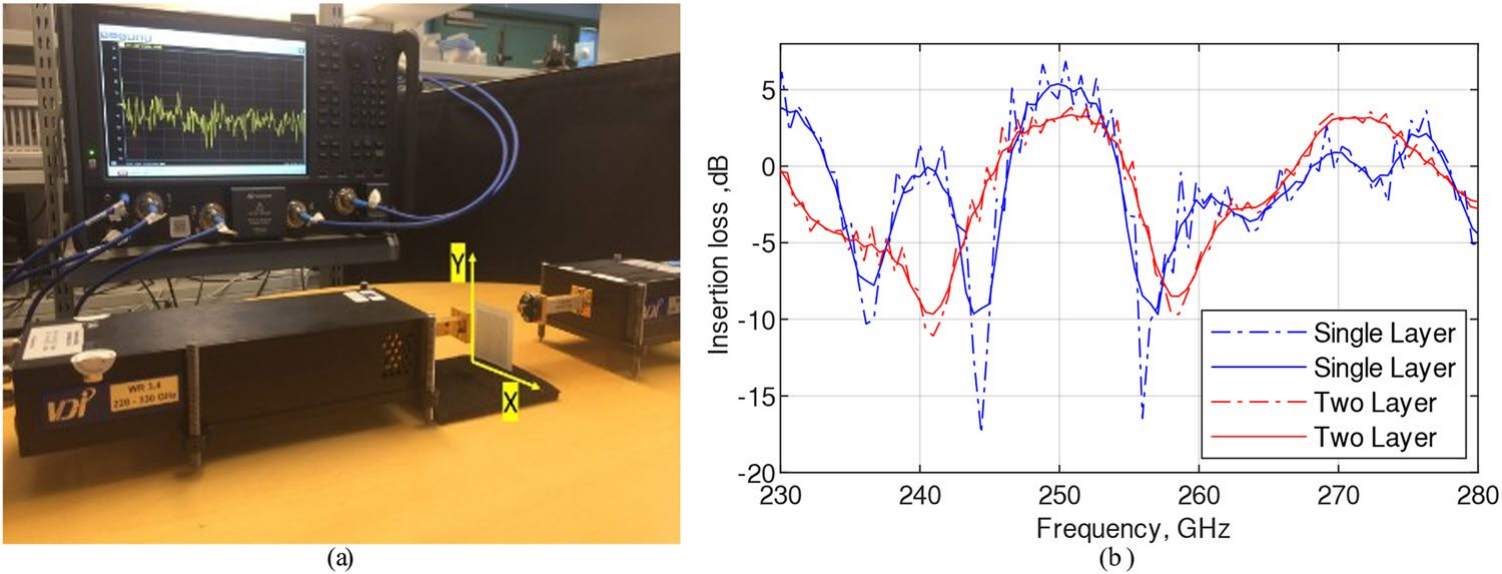
Insertion loss was measured by placing the filter between the waveguide and horn antenna as in (**a**) and (**b**) shows the measured signal in dashed line. The solid line is smoothed version.

A microwave measurement system consisting of a PNA (operation frequency 67 GHz) and a pair of VDI extenders (Tx and Rx with operation frequency from 220 to 330 GHz) was used to measure the S-parameters, as shown in Fig. 8a. The Tx was connected to a rectangular waveguide (WR3.4) and a standard horn antenna (TE mode) was connected to the Rx. The FSS filter was located in the middle of the beam and the measured S-parameters are shown in Fig. 8b. The results show insertion losses with and without smoothing, solid and dashed line. Measured insertion losses for both cases at 250 GHz were ~ 3 dB. Bandwith for a single layer was ~ 7 GHz and for a double layer it was 9 GHz.

## Comparison between the simulation and experiments

**Figure 9:**
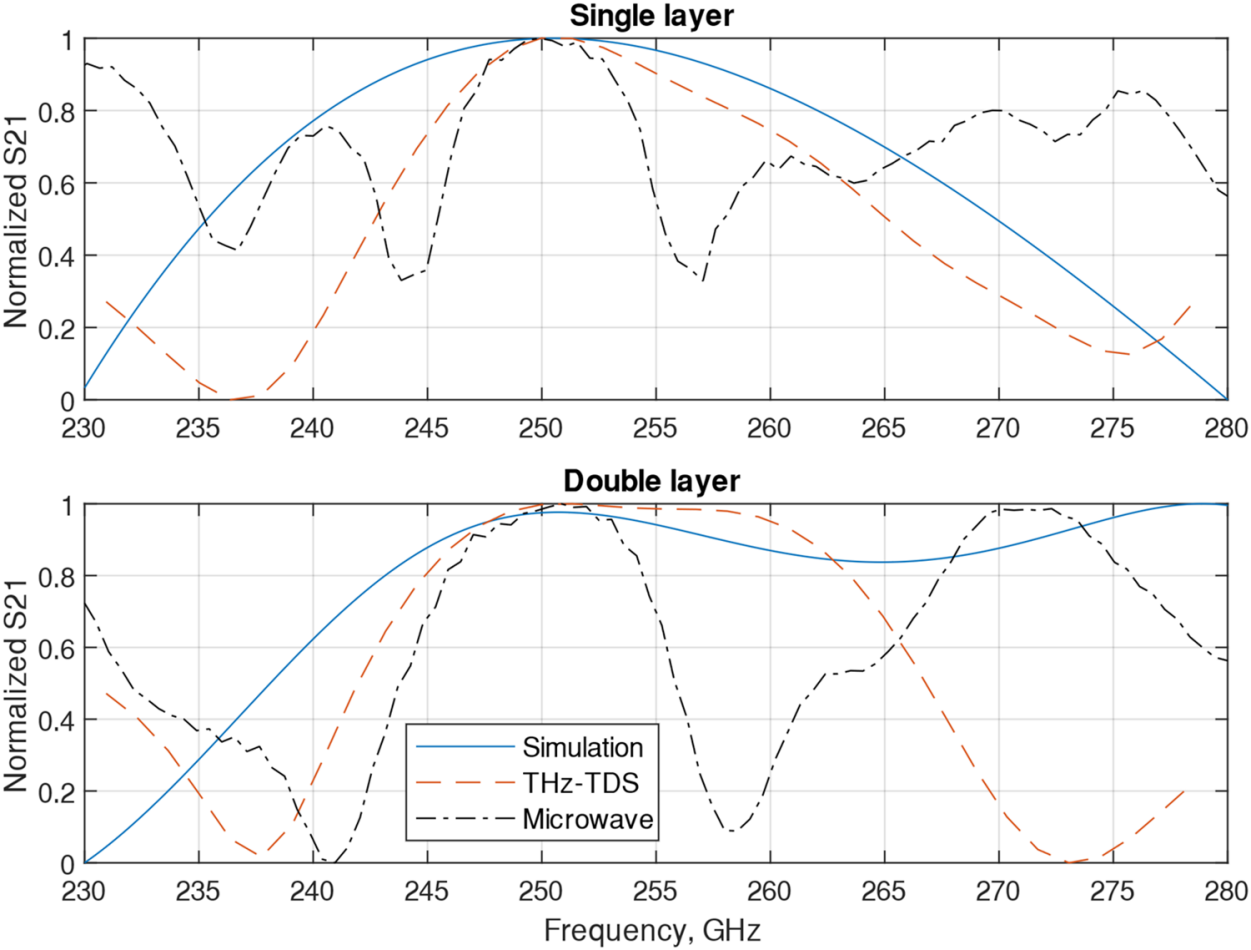
Comparison of the simulation, THz-TDS, and microwave normalized results.

The FSS simulation, THz-TDS, and microwave measurements normalized results are presented in Fig. 9. Simulations were done using a Floquet mode simulation which used either a TE or TM planewave as a propagating wave, the THz-TDS use the focused pulse propagating in free space and the microwave system a wave guide and a horn antenna (both TE mode antennas).

For a single layer, the simulation bandwidth was around 20 GHz, 10 GHz for THz-TDS and 7 GHz for the microwave system. For two layers, the simulation bandwidth was around 27 GHz, 16 GHz for THz-TDS and 9 GHz for the microwave system. Simulation for the single layer case showed a wider bandwidth compared to the THz-TDS and microwave system. In the microwave results, grating lobes are clearly visible in the single- and double-layer cases. Despite the differences in the simulations and measurements, they all conclude the same thing, a cascaded FSS filter on a PCB does work in both simulations and measurements in the Sub-THz frequency band.

## Conclusion

This work presented an FSS filter consisting of SRR’s on PCB (RT5880) for a 250 GHz center frequency. The FSS layer was designed with a full wave simulation for TE mode and it was predicted to have 27 GHz bandwidth when two FSS layers were at a distance of 1.367 mm from each other. It was noted that changing the layers’ distance impacted on the bandwidth. On increasing the spacing the bandwidth was decreased and vice versa. This allows another way to control the bandwidth as only the distance of the layers needs to be changed. Also, the inter element spacing impacted on the bandwidth; having too large a spacing caused the bandwidth to decrease. Additionally, phase was impacted by a second layer as well. At 250 GHz a 27 degree shift from a single layer level was observed.

The filter was fabricated on RT5880 using a lithography method and the resulting filter had 32 × 30 unit cells on an area ~ 3 × 3 cm^2^. The cascaded filter was measured with THz-TDS and a microwave system and the measured bandwidth was 16 GHz and 9 GHz, respectively. Additionally, in the THz-TDS measurements a time delay and signal transmission was analyzed. The time delay was 0.1 ps and 0.5 ps and transmission 0.9 and 0.43 for single and double layer, respectively. Variations between the results have been considered to come from different wave shapes, beam widths and different measurement system geometries. Cascaded filters could be one of the potential solutions to increase the filter bandwidth in the future.

## Data Availability

The data that support the findings of this study are available from the corresponding author, A. G, upon reasonable request.
